# Exploring the Dynamics of Sleep Deprivation: Insights into Complete Blood Count and Coagulation Parameters in a Case-Control Study

**DOI:** 10.1155/2024/1766578

**Published:** 2024-04-18

**Authors:** Abd Elhadi Agena, Leena Mirghani, Abdirasak Ali Mude

**Affiliations:** ^1^Faculty of Medical Laboratory Sciences, Elimam Elmahdi University, Kosti 249, Sudan; ^2^Faculty of Medical Laboratory Sciences, Hematology Department, Al-Neelain University, Khartoum 249, Sudan; ^3^Faculty of Medicine and Health Sciences, Simad University, Mogadishu 252, Somalia

## Abstract

**Background:**

The lack of preceding research in Sudan emphasizes the importance of this study, which contributes critical data to the global understanding of sleep-related health effects. This study investigates the complex relationship between sleep deprivation and blood-related factors, particularly focusing on full blood count and coagulation parameters.

**Methods:**

From January to March 2022, a case-control study was conducted in Kosti, Sudan. A control group of 11 healthy 23–33-year-olds (6 men and 5 women) had regular sleep patterns. Six men and five women ages 23–33 were chosen for this sleep-deprived case study. The case group was deprived of sleep from 7:00 p.m. to 7:00 a.m. for three days and allowed to sleep normally during the day. Daily at 7:00 a.m., antecubital vein blood was drawn. The ACL 7000 coagulation analyzer and Sysmex fully automated hematology analyzers were used for coagulation and whole blood count analysis. Data analysis included descriptive and inferential approaches like the Mann–Whitney *U* test for group comparisons.

**Results:**

The study found no significant differences in total white blood cell counts reported between case and control groups (*p*=0.898). The case group had a substantial drop in lymphocyte counts on day 3 (*p*=0.016). The third day showed significant differences in neutrophil and eosinophil levels (*p*=0.003 and 0.000, respectively). The difference in hemoglobin and hematocrit on day 3 was statistically significant (*p*=0.023). Platelet counts were stable. Both groups' prothrombin times were unaffected. On all three days, groups had significant differences in activated partial thromboplastin time (APTT) (*p*=0.004). Therefore, the intrinsic coagulation system may have changed.

**Conclusion:**

This study demonstrates the complex link between sleep deprivation, coagulation indicators, and complete blood count. Monitoring blood indicators in poor sleep helps explain fundamental mechanisms and medicinal implications.

## 1. Introduction

Sleep plays a vital role in maintaining good health, as its timing, length, and quality affect metabolic control, emotion management, performance, memory consolidation, brain recovery, and learning [1]. Additionally, it plays a crucial function in enhancing health by impacting the susceptibility to infectious diseases, severe medical conditions, and mental well-being [2]. Moreover, sleep is associated with the control of endocrine activity, which is crucial for preserving a balanced body composition [3]. Studies have demonstrated that implementing interventions such as brief periods of rest and moderate physical activity in older individuals can enhance the quality of sleep, hence resulting in improved mental and physical well-being [4]. The cardiovascular reactions to both normal and troubled sleep are intricate, encompassing dynamic alterations in autonomic, electroencephalogram (EEG), and hemodynamic reactions [5]. Hence, placing sleep as a top priority is vital for upholding one's general well-being.

Multiple studies have consistently demonstrated that a lack of sleep can have notable impacts on blood-related factors and the body's ability to clot, potentially elevating the likelihood of developing cardiovascular conditions. A study conducted by Liu revealed that sleep deprivation can result in increased levels of white blood cells and neutrophils, as well as reduced prothrombin and activated partial thromboplastin durations, indicating a state of hypercoagulability [6]. Wang discovered a direct correlation between the amount of time spent sleeping and the levels of hematocrit in the blood. Longer sleep was found to increase both hematocrit and hemoglobin levels [7].

Mejri discovered that partial sleep deprivation had no significant impact on red blood cell count, hemoglobin levels, or hematocrit during intermittent activity [8]. Meier-Ewert's research provided more evidence that sleep deprivation can increase levels of high-sensitivity C-reactive protein, a biomarker of inflammation linked to cardiovascular risk [9]. Prather also emphasized the gender-specific influence of inadequate sleep quality on inflammatory markers, noting that women exhibited notable elevations in these markers [10]. Holmer's research revealed that sleep deprivation can hinder the proper functioning of endothelial cells, hence increasing the likelihood of developing cardiovascular disease [11]. These findings emphasize the significance of investigating the impact of sleep deprivation on blood-related factors and blood clotting patterns, especially in relation to cardiovascular well-being.

Previous studies on sleep deprivation (SD) and biological responses (e.g., hematological) are unclear due to information gaps. Several studies show that partial SD and total SD increase WBC and their subpopulations. Some saw no sleep loss or reduction in these measurements, and HCT and RBC seem unaffected by SD [8].

The lack of preceding research in Sudan emphasizes the importance of this study, which contributes critical data to the global understanding of sleep-related health effects. Conducting the initial study on the dynamics of sleep deprivation, this research endeavors to fill the current void in scientific investigation within Sudan. By concentrating on coagulation parameters and complete blood count, this study aims to offer significant contributions to the understanding of the physiological consequences of sleep deprivation within the context of Sudan.

## 2. Materials and Methods

### 2.1. Study Design, Area, and Duration

**Figure 1:**
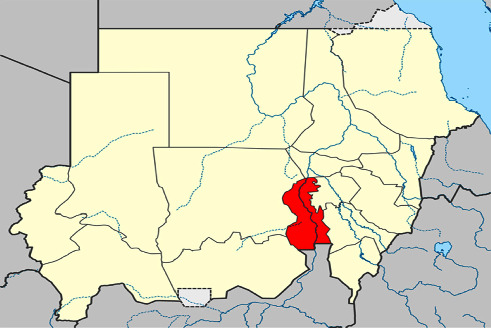
Kosti, a Sudanese city, is located south of Khartoum on the west bank of the White Nile and is connected to Rabak by a bridge.

This study utilized a case-control study design. Kosti, situated south of Khartoum, the capital of Sudan, is a significant urban center. It is situated on the western branch of the White Nile river, in direct opposition to Rabak, the capital of the White Nile state, with which it shares a bridge (Figure 1). The estimated population of Kosti in 2012 was 345,068 individuals. Data were collected from January to March 2022.

### 2.2. Subjects

A total of 22 healthy volunteers were recruited ranging in age from 23 to 33, with 11 individuals assigned to each group (one group serving as the control, consisting of individuals with regular sleep patterns, and the other group as the case, consisting of individuals who were sleep deprived). Every participant provided informed written consent before taking part in this study. The inclusion criteria for participants were a state of good health and the absence of medicines for at least two months. Patients with hepatitis, other serious illnesses (including sleep problems), or continuing infections (fever, inflammation, or indirect indicators) were not included in the study. Neither were patients experiencing stressful life events at the time. Women in the study were not in a specific menstrual phase. Throughout the investigation, nutritious breakfast, lunch, and dinner were served at acceptable times, and caffeine was restricted. Staff provided minimal social engagement to keep patients alert at all times. Participants knew they could leave at any time without risk. The investigation would have been stopped if more than two subjects withdrew. The University of El-Imam El-Mahdi approved this study protocol.

## 3. Method

### 3.1. Collection of Samples

Blood samples were obtained using EDTA and trisodium anticoagulant tubes. EDTA is used for complete blood counts, while trisodium is used for coagulation assays, such as PT, INR, and APTT. The samples were analyzed within a period of 4 hours.

### 3.2. Examination of Complete Blood Count

The impedance approach quantified alterations in electrical resistance caused by the passage of blood cells through a precisely calibrated aperture. The Sysmex analyzer was deployed to do a complete blood count (CBC) analysis [12].

### 3.3. Examination of Coagulation

The ACL 7000 methodology employed spectrophotometry to evaluate coagulation variables through the execution of PT and APTT tests [13].

### 3.4. Data Analysis

A comprehensive analysis of the data included both descriptive and inferential techniques. The normality of the data was assessed through the Shapiro–Wilk test. As the data did not follow a normal distribution, the median and interquartile range (IQR) were calculated for continuous variables. To assess differences of various blood cell indices among cases and controls, the Mann–Whitney *U* test was employed. Statistical significance was determined at a threshold of *p* ≤ 0.05 with a 95% confidence interval. All statistical analyses were conducted using IBM's SPSS software, version 27.0.1, ensuring robust and reliable statistical evaluations.

## 4. Results

**Figure 2:**
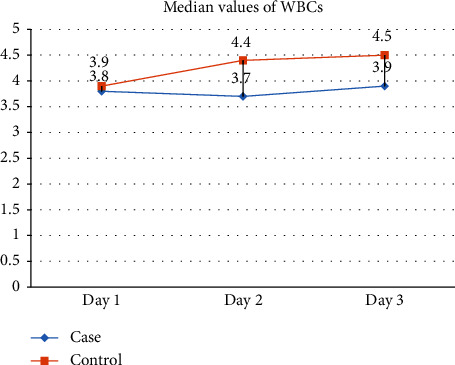
Showed the median values of WBCs in cases and control group across three days. Values were consistently lower among the cases on day 2 and 3.

The impact of sleep deprivation on white blood cell counts yielded compelling results. Across the three days, there were no significant differences in total white blood cell counts between the Case and Control groups, with *p* values consistently exceeding 0.05. However, a noteworthy finding emerged concerning lymphocyte counts, revealing significant decrease on Day 3 (*p*=0.016), indicating potential immune system susceptibility during extended sleep deprivation. In contrast, monocyte counts did not exhibit significant differences between the groups (*p* values >0.05). Interestingly, eosinophil counts showed significant decrease on the third day (*p* < 0.05), indicating a notable impact of sleep deprivation on eosinophil levels. Further analysis revealed significant decrease in neutrophil counts on Day 3 (*p*=0.003) (shown in Table 1 and Figure 2).

**Figure 3:**
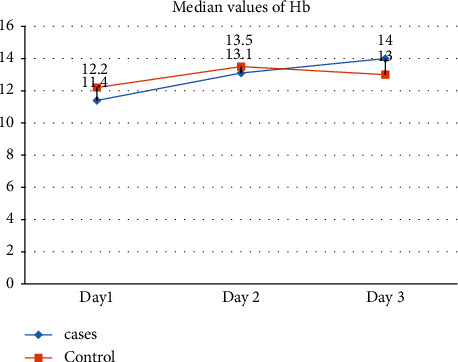
Showed that the hemoglobin levels showed interesting trends. On day 1 and 2 hemoglobin levels were higher among controls while on day three levels were higher among cases.

No significant differences in hemoglobin levels were observed on Day 2 (*p*=0.847). However, on Day 1, there was a significant decrease in hemoglobin levels in the case group compared to the control group (*p*=0.040). On Day 3, a significant increase in hemoglobin levels was noted in the case group (*p*=0.023). Day 1 and Day 2 did not show significant differences in HCT levels (*p*=0.056 and *p*=0.699, respectively). On Day 3, there was a significant increase in HCT levels in the Case group compared to the Control group (*p*=0.023). No significant differences in MCV were observed on Day 1 (*p*=0.243) and Day 2 (*p*=0.116). On Day 3, a significant increase in MCV was noted in the Case group (*p*=0.040). No significant differences were observed in MCH levels on Day 1 (*p*=0.133), Day 2 (*p*=0.243), and Day 3 (*p*=0.300). No significant differences in MCHC were observed on Day 1 (*p*=0.088) and Day 2 (*p*=0.300). Day 3 also showed no significant differences in MCHC levels (*p*=0.797). No significant differences in RDWC were observed on Day 1 (*p*=0.797) and Day 2 (*p*=1.000). On Day 3, there was a significant decrease in RDWC in the Case group (*p*=0.047) compared to control group. No significant differences were observed in RDWS on Day 1 (*p*=0.652), Day 2 (*p*=0.300), and Day 3 (*p*=0.365) (shown in Table 2 and Figure 3).

**Figure 4:**
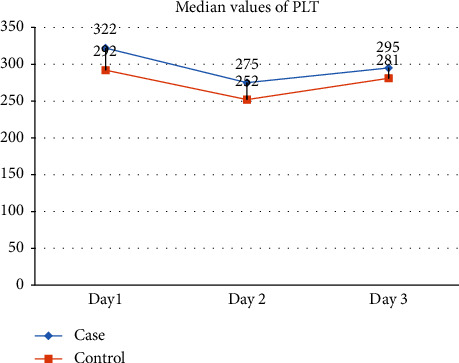
Showed that the platelet levels were consistently higher among cases on all the three days.

There were no significant variations in platelet count when exploring the impact of sleep loss on platelet parameters. On Day 1, the case group exhibited a median of 322.00 compared to the control group's 292.00, yielding a nonsignificant *p* value of 0.478. Days 2 and 3 similarly showed no statistically significant differences, with *p* values of 0.652 and 0.270, respectively. Additional platelet indices, including mean platelet volume, platelet distribution width, plateletcrit, and platelet large cell ratio, also demonstrated no significant variations (shown in Table 3 and Figure 4).

**Figure 5:**
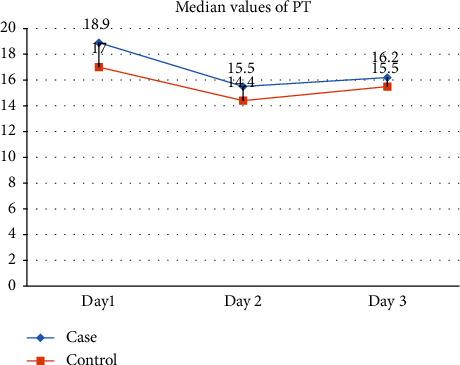
Showed that PT was higher on all three days among cases as compared to controls however, the difference between the PT levels between the two groups narrowed on day 3.

**Figure 6:**
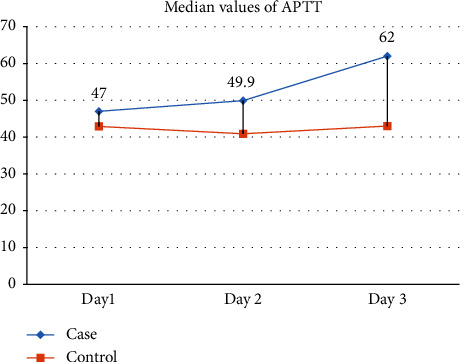
Showed that the APTT level although higher among the cases. This difference increased on day 3.

On Day 1, the case group exhibited a median Prothrombin Time (PT) of 18.90, while the control group had a median of 17.00, yielding a nonsignificant *p* value of 0.438. Days 2 and 3 showed *p* values of 0.193 and 0.088, respectively, indicating no statistically significant differences. INR values on Day 1, Day 2, and Day 3 demonstrated *p* values of 0.748, 0.193, and 0.243, respectively, suggesting no significant differences between the case and control groups. APTT values showed significant prolongation on all three days. On Day 1, the case group had a median APTT of 47.00, while the control group had 42.90 with a *p* value of 0.004. Days 2 and 3 also exhibited significant differences with *p* values of 0.023 and 0.005, respectively (shown in Table 4 and Figures 5 and 6).

## 5. Discussion

This study explores the complex relationship between lack of sleep and the blood-related factors, revealing subtle impacts on different components. The data displayed in Tables 1–4 illuminate the intricate connections, unveiling both unexpected consistencies and notable deviations.

In contrast to the anticipated results from prior research, our analysis revealed no substantial disparities in the overall count of white blood cells (WBC) between the sleep-deprived Case group and the Control group over the course of three days. The continuous *p* values exceeding 0.05 imply that the overall WBC levels remain stable, which contradicts the idea of a widespread shift in the WBC population due to poor sleep. An interesting finding was the notable reduction in lymphocyte counts on Day 3 in the Case group (*p*=0.016). Lymphocytes, essential constituents of the immune system, have a critical function in safeguarding the body from infections. The decreased lymphocyte numbers indicate a susceptibility to infections while experiencing extended sleep deprivation, emphasizing a particular element of immune function that is impacted by inadequate sleep. However, the monocyte counts showed no notable disparities between the Case and Control groups, suggesting that sleep loss may not have a significant influence on this category of white blood cells. The stability observed indicates that sleep loss has minimal impact on monocytes, which play a role in immunological responses and inflammation. The study found a statistically significant decrease in eosinophil counts on the third day (*p* < 0.05), indicating a significant effect of sleep deprivation on these cells. Eosinophils play a critical role in fighting parasite infections and allergic reactions. Furthermore, the changes in the number of neutrophils on Day 3 (*p*=0.003) underscore the active reaction of the immune system to extended sleep deprivation, thus emphasizing the distinctiveness of the immunological response towards various cell types. The notable disparities in the percentages of lymphocytes and neutrophils on Day 3 (*p*=0.005 and *p* < 0.001, respectively) offer a more profound understanding of the composition of the white blood cell population. These alterations imply a possible change in the equilibrium among many types of immune cells under prolonged sleep deprivation, indicating an intricate and diverse reaction. These results align with existing literature, emphasizing the complex interplay between sleep and immune function [6, 14].

Table 2 demonstrates notable fluctuations in hemoglobin levels between Day 1 and Day 3, indicating a responsive and changing behavior of the hematological system in response to sleep loss. The observed rise in hemoglobin levels on Day 3 may suggest compensatory mechanisms to uphold the capacity to transport oxygen, demonstrating the body's ability to adjust in situations of inadequate sleep. Notably, hematocrit levels exhibited a substantial increase on Day 3, indicating possible alterations in blood viscosity. Although these modifications were noticed, no substantial disparities were detected in other red blood cell indices. These findings confirm prior research that suggests a connection between inadequate sleep and hematocrit levels. These findings echo previous studies indicating associations between poor sleep and hematocrit levels [14–17].

Contrary to expectations, the lack of sleep did not cause any notable variations in the number of platelets or different platelet measurements, as seen in Table 3. The platelet parameters exhibit stability, indicating that sleep deprivation has minimal impact on this essential element of the hematological system, hence preserving its vital function in the process of blood clotting. Table 4 demonstrates substantial modifications in APTT values throughout all three days, suggesting variations in the intrinsic coagulation system. These findings indicate that lack of sleep might impact the body's blood clotting processes, which in turn may increase the likelihood of bleeding or hypercoagulable states. The practical significance of these discoveries emphasizes the necessity of monitoring blood clotting factors in persons undergoing sleep deprivation. Although there were no notable disparities in prothrombin time (PT) and international normalized ratio (INR), their therapeutic importance requires careful observation. Under situations of sleep deprivation, there is a notable emphasis on the stable extrinsic coagulation pathway, highlighting the importance of conducting thorough evaluations of coagulation components, aligning with previous studies linking sleep loss to changes in coagulation profiles [6, 7, 18–21].

Limitations of this study include sample bias, short observation duration, lack of attention to individual sleep patterns, use of a single measurement method, uncontrolled external factors, impact of participant awareness, and insufficient investigation of mechanisms. To fully understand and plan for future research, it is essential to address these limitations. For a more complex understanding of the hematological effects of sleep deprivation, future research could use diversified samples, longitudinal analyses, different measurement techniques, control of external variables, reduction of participant awareness bias, and exploration of mechanistic pathways.

In conclusion, this study offers significant understanding into the intricate correlation between sleep deprivation and immunological and hematological factors. The observed fluctuations in lymphocyte, eosinophil, and neutrophil counts, as well as changes in coagulation factors, highlight the necessity for additional investigation to unravel the underlying mechanisms and clinical implications. Gaining a comprehensive understanding of how sleep deprivation affects the immunological and hematological function is crucial to design specific ways to reduce potential health problems that may arise from inadequate sleep.

## Figures and Tables

**Table 1:** Effect of sleep deprivation on total white blood cells and differential counts.

Parameters		Groups				*p* value
		Case		Control		
		Median	IQR (Q_3_–Q_1_)	Median	IQR (Q_3_–Q_1_)	
WBC (×10^9^/L)	Day 1	3.80	3	3.90	2.20	0.898
	Day 2	3.70	1.70	4.40	3	0.478
	Day 3	3.90	0.80	4.50	1.70	0.300

Lymphocyte (×10^9^/L)	Day 1	1.60	1.40	1.80	0.60	1.000
	Day 2	1.70	0.70	2.10	0.80	0.699
	Day 3	1.80	0.30	1.50	0.30	0.016

Neutrophil (×10^9^/L)	Day 1	1.50	1.20	1.40	1.30	0.898
	Day 2	1.70	1	1.50	1.80	0.748
	Day 3	1.50	0.50	2.50	1.40	0.003

Monocyte (%)	Day 1	7.50	2.80	8.80	4.10	0.243
	Day 2	7.10	2	8.00	4.10	0.300
	Day 3	7.60	3.80	4.20	5.70	0.652

Eosinophil (%)	Day 1	8.30	6.80	6.00	6.20	0.076
	Day 2	7.10	4.10	9.30	8.30	0.519
	Day 3	8.40	2.30	2.70	1.40	0.000

Basophil (%)	Day 1	0	0	0	0	—
	Day 2	0	0	0	0	—
	Day 3	0	0	0	0	—

**Table 2:** Effect of sleep deprivation on hemoglobin and red blood cell indices.

Parameters		Groups				*p* value
		Case		Control		
		Median	IQR (Q_3_–Q_1_)	Median	IQR (Q_3_–Q_1_)	
Erythrocyte	Day 1	4.58	0.51	4.55	0.38	0.232
	Day 2	5.24	0.7	4.90	1.32	0.242
	Day 3	5.38	0.57	5.15	0.7	0.232

Hemoglobin (g/dl)	Day 1	11.40	2.90	12.20	1.10	0.040
	Day 2	13.10	1.50	13.50	2.40	0.847
	Day 3	14.00	1.80	13.00	1.80	0.023

Hematocrit (%)	Day 1	36.60	3.40	37.70	2.20	0.056
	Day 2	41.30	3.50	42.90	7.60	0.699
	Day 3	44.30	4.30	40.60	4.60	0.023

MCV (fl)	Day 1	82.70	18.10	81.80	6.80	0.243
	Day 2	78.90	5.60	82.10	8.30	0.116
	Day 3	81.00	4.30	78.70	3.60	0.040

MCH (pg)	Day 1	24.90	8.20	27.20	3.70	0.133
	Day 2	25.00	1.50	25.70	3.10	0.243
	Day 3	25.50	1.70	25.10	0.80	0.300

MCHC (%)	Day 1	30.50	3.50	32.40	2.20	0.088
	Day 2	31.80	2.70	31.00	2.10	0.300
	Day 3	31.60	2.70	31.60	1.90	0.797

RDW-CV (%)	Day 1	11.70	4	12.60	1.50	0.797
	Day 2	12.00	1.70	12.20	0.60	1.000
	Day 3	12.00	0.70	12.60	0.80	0.047

RDW-SD (fl)	Day 1	37.40	2.80	37.10	3.80	0.652
	Day 2	35.50	1	36.60	2.50	0.300
	Day 3	35.50	0.80	36.30	3.30	0.365

MCV: mean cell volume; MCH: mean cell hemoglobin; MCHC: mean cell hemoglobin concentration; RDW-SD: red cell distribution width standard deviation; RDW-CV: red cell distribution width coefficient variation.

**Table 3:** Effect of sleep deprivation on platelet counts and platelet indices.

Parameters		Groups				*p* value
		Case		Control		
		Median	IQR (Q_3_–Q_1_)	Median	IQR (Q_3_–Q_1_)	
Platelet (×10^9^/L)	Day 1	322.00	239	292.00	46	0.478
	Day 2	275.00	124	252.00	165	0.652
	Day 3	295.00	50	281.00	103	0.270

MPV (fl)	Day 1	10.30	1.80	9.50	0.70	0.217
	Day 2	11.00	3.70	9.70	1.20	0.171
	Day 3	9.40	2.60	9.60	1.60	0.478

PDW (fl)	Day 1	13.40	5	12.30	1.30	0.217
	Day 2	14.80	7.30	12.40	2.50	0.300
	Day 3	11.60	4	11.90	2.80	0.401

PCT (%)	Day 1	0.33	0.16	0.27	0.10	0.193
	Day 2	0.25	0.9	0.25	0.14	0.797
	Day 3	0.27	0.8	0.26	0.07	0.606

PLCR (%)	Day 1	31.80	15.50	24.80	5.60	0.332
	Day 2	36.30	27.10	25.60	10.20	0.171
	Day 3	24.40	19.10	25.30	16	0.699

MPV: mean platelet volume; PDW: platelet distribution width; PLCR: platelet large cell ratio; PCT: plateletcrit.

**Table 4:** Effect of sleep deprivation on coagulation factors.

Parameters		Groups				*p* value
		Case		Control		
		Median	IQR (Q_3_–Q_1_)	Median	IQR (Q_3_–Q_1_)	
PT (sec)	Day 1	18.90	4	17.00	6.70	0.438
	Day 2	15.50	3.80	14.40	4.10	0.193
	Day 3	16.20	9.40	15.50	2.20	0.088

INR (%)	Day 1	1.34	1.36	1.20	0.54	0.748
	Day 2	1.05	0.32	0.96	0.27	0.193
	Day 3	1.11	0.83	1.08	0.39	0.243

APTT (sec)	Day 1	47.00	12.30	42.90	7	0.004
	Day 2	49.90	12.90	40.90	3.50	0.023
	Day 3	62.00	34.60	43.00	4.10	0.005

PT: prothrombin time; APTT: activated partial thromboplastin time; INR: international normalized ratio.

## Data Availability

The data used to support the findings of this study are available from the corresponding author upon request.
